# Efficacy of regorafenib combined with PD-1 inhibitors in elderly patients with advanced metastatic colorectal cancer

**DOI:** 10.1186/s12877-022-03637-9

**Published:** 2022-12-21

**Authors:** Beibei Chen, Huichen Zhao, Jinxi Huang, Huifang Lv, Weifeng Xu, Caiyun Nie, Jianzheng Wang, Jing Zhao, Yunduan He, Saiqi Wang, Xiaobing Chen

**Affiliations:** 1 Department of Medical Oncology, The Affiliated Cancer Hospital of Zhengzhou University, Henan Cancer Hospital, Zhengzhou, 450008 Henan Province China; 2 Zhengzhou Key Laboratory for Precision Therapy of Gastrointestinal Cancer, Zhengzhou, 450008 Henan Province China; 3 Department of Gastrointestinal Surgery, The Affiliated Cancer Hospital of Zhengzhou University, Henan Cancer Hospital, Zhengzhou, 450008 Henan Province China

**Keywords:** Colorectal cancer, Elderly patients, Immunotherapy, Programmed cell death protein 1, Regorafenib

## Abstract

**Objective:**

This is the first clinical study that wants to investigate the treatment patterns, clinical outcomes, and prognostic factors of regorafenib plus PD-1 inhibitors therapy in Chinese elderly patients with advanced colorectal cancer.

**Methods:**

A cohort of metastatic colorectal cancer patients 60 years or older who received treatment with regorafenib combined with PD-1 inhibitors was included in our analysis. The endpoints included overall survival (OS), progression-free survival (PFS), and prognostic factors.

**Results:**

In total, 24 patients were enrolled with the median age of 68 years, and 62.5% were female. The median OS and PFS were 15.03 months (95% CI 7.0–23.0) and 4.0 months (95% CI 1.8–6.2), respectively. The objective response rate was 8.3%, and the disease control rate was 70.8%. Patients previously treated with regorafenib had a longer median PFS than those without (6.3 versus 2.8 months). In terms of final daily doses, it showed a trend toward better PFS (median PFS was 10.0 months) in high-dose group (daily dose above 80 mg of regorafenib) compared to low-dose group (daily dose no more than 80 mg of regorafenib) (median PFS was 3.5 months).

**Conclusions:**

This real-world evidence confirms that Chinese elderly patients with advanced colorectal cancer may benefit from the treatment of regorafenib combined with PD-1 inhibitors, similarly with this combination therapy strategies in all age patients.

## Introduction

Colorectal cancer (CRC), as one of the most prevalent types of cancer in China, is still a leading cause of cancer-related mortality [1]. Based on a multicenter retrospective study from China, the median age of metastatic CRC (mCRC) cases at diagnosis was 58 years, and almost half of mCRC patients were aged 60 years or older [2]. Therapeutic decisions involving elderly patients are a serious issue in oncology because this group is characterized by a higher incidence of significant co-morbidities (cardiovascular disorders, metabolic disorders, and liver disorders), decreased regenerative capacity of bone marrow (higher incidence and intensity of hematological complications of chemotherapy) as well as worse general performance [3]. Making all anticancer drugs available to elderly patients with mCRC is important to achieve the maximal benefit for long-term survival and maintain their quality of life.

Regorafenib, an oral small-molecule multi-kinase inhibitor, has demonstrated significantly improved survival in two randomized, double-blind, placebo-controlled phase 3 trials (CORRECT and CONCUR) [4, 5]. Therefore, it is recommended as a standard third- or later-line therapy for refractory mCRC by the Chinese Society of Clinical Oncology (CSCO) guideline and other international guidelines [6–8]. Immune checkpoint inhibitors have shown promising therapeutic outcomes in advanced colorectal cancer with bearing mismatch repair-deficiency/microsatellite instability-high (dMMR/MSI-H) tumors in recent years [7–10]. However, patients with mismatch repair-proficient/microsatellite stable (pMMR/MSS) colorectal cancer, who account for 95% of advanced colorectal cancer, could not benefit from this approach [11].

Recent data suggest a possible synergic effect between regorafenib and immune checkpoint inhibitors, as has been shown in the REGONIVO trial, which reported a response rate of 33% and median progression-free survival of up to 7.9 months in a cohort of 24 Japanese patients with MSS treatment-refractory metastatic colorectal cancers [12]. In the REGOTORI trial, which produced similar results, the objective response rate (ORR) was 15.2%, and median overall survival was 15.5 months in patients with refractory pMMR/MSS mCRC [13]. However, further research should be conducted to address this therapeutic strategy applied to elderly Chinese patients. This study aimed to investigate the treatment patterns, clinical outcomes, and prognostic factors of regorafenib plus PD-1 inhibitors therapy in Chinese elderly patients with advanced colorectal cancer.

## Materials and methods

### Study population

We retrospectively reviewed the patients treated with regorafenib plus PD-1 inhibitors for unresectable mCRC in Henan Cancer Hospital (China) from January 2019 to July 31, 2021.

Patients were eligible for participation if they were 60 years of age or older and had histologically or cytologically confirmed mCRC. Patients were required to have failed to respond to all the available systemic agents, including fluoropyrimidine, oxaliplatin, irinotecan, bevacizumab, and cetuximab when applicable, and received at least one cycle of regorafenib plus PD-1 inhibitors. Besides, the medical record should be complete and legible. Patients who met any of the following criteria at the time of screening will be excluded: other histological types instead of adenocarcinoma of the colon or rectum; at stage I ~ III according to TNM staging system; Eastern Cooperative Oncology Group (ECOG) performance status of 3 or more. This work was approved by the ethics committee of Henan Cancer Hospital (Approval number: 2020103005). Patients were followed up by telephone questionnaires every three months until death or this study cutoff date (July 31, 2021).

### Treatment and evaluation of therapeutic efficacy

Regorafenib was prescribed at 40, 80, 120, or 160 mg daily from day 1 to day 21 of each 28-day cycle. Depending on the patient’s tolerability, the daily dose was allowed at the discretion of treating physicians. All patients received immune checkpoint inhibitors on the first day of regorafenib treatment, PD-1 inhibitors were used according to the recommended doses: nivolumab 240 mg every two weeks, camrelizumab 200 mg every two weeks, sintilimab 200 mg every three weeks, toripalimab 240 mg every three weeks, pembrolizumab 200 mg every three weeks, tislelizumab 200 mg every three weeks.

Demographics, disease features, and therapy information of patients were documented at baseline examinations. Tumors response was evaluated every 2 or 3-cycle treatment of PD-1 inhibitors, according to the Response Evaluation Criteria in Solid Tumors (RECIST) Version 1.1.

The primary endpoint was overall survival (OS), which was defined as the duration from treatment to death as a result of any cause. Other endpoints included progression-free survival (PFS), disease-control rate (DCR), and objective response rate (ORR). Progression-free survival (PFS) was defined as the duration from treatment to the first documented disease progression or death. The objective response rate (ORR) was defined as complete response (CR) plus partial response (PR), and the disease-control rate (DCR) was defined as CR plus PR together with stable disease (SD). Adverse Events were graded and analyzed according to National Cancer Institute Common Terminology Criteria for Adverse Events (NCI-CTCAE), Version 4.03.

### Statistical analysis

All statistical analysis was performed using IBM SPSS Statistics for Windows Version 25.0 (IBM Corp., Armonk, New York, USA). Quantitative data were displayed as mean with standard deviation (SD), and qualitative data were expressed as a number with percentage [No. (%)]. Survival data were analyzed using the Kaplan–Meier method and compared by log-rank test. *P* value < 0.05 was considered statistically significant. 

## Results

### Study participants’ characteristics

At a median follow-up of 16.2 months, a total of 24 patients were enrolled. Nine of these patients (37.5%) were male; the median age was 68.0 years. The primary tumor site was the right-side colon in 33.3% (*n* = 8) of patients, the left-side colon in 29.2% (*n* = 7), and the rectum in 37.5% (*n* = 9). The main metastatic sites were the liver and/or lung (91.7%), only the lung (25.0%), only the liver (29.2%), distal lymph nodes (45.8%), and peritoneum (16.7%). Other baseline characteristics of the patients are presented in Table 1.

**Table 1:** Baseline demographic and clinical characteristics of 24 mCRC patients

Characteristics	*N* = 24 patients n (%)
Age (year)	
Median (range)	68(61–77)
Gender	
Male	9 (37.5%)
Female	15 (62.5%)
ECOG performance status	
0	5 (20.8%)
1	16 (66.7%)
2	3 (12.5%)
Primary tumor location	
Colon	15 (62.5%)
Right-side	8 (33.3%)
Left-side	7 (29.2%)
Rectum	9 (37.5%)
Whether the primary tumor is resected	
Resected	21 (87.5%)
Not resected	3 (12.5%)
Type of metastasis	
With liver or lung metastasis	22 (91.7%)
With only liver metastasis	6 (25.0%)
With only lung metastasis	7 (29.2%)
With liver and lung metastasis	9 (37.5%)
With bone metastasis	3 (12.5%)
With brain metastasis	1 (4.2%)
With distant lymph nodes metastasis	11 (45.8%)
With peritoneum metastasis	4 (16.7%)
With other organs metastasis	2 (8.3%)
Previous lines of chemotherapy	
Two lines	13 (54.2%)
Three or more lines	11 (45.8%)
Previous targeted therapy	
Bevacizumab	15 (62.5%)
Cetuximab	3 (12.5%)
With Bevacizumab and Cetuximab	3 (12.5%)
Without previous targeted therapy	6 (25.0%)
Combination immunization agents	
Sintilimab	12 (50.0%)
Carrelizumab	6 (25.0%)
Nivolumab	2 (8.3%)
Toripalimab	2 (8.3%)
Pembrolizumab	1 (4.2%)
Tislelizumab	1 (4.2%)
Whether previous exposure to regorafenib	
Exposure to regorafenib	8 (33.3%)
No exposure to regorafenib	16 (66.7%)
Whether with the local treatment	
With the local treatment	3 (12.5%)
Without the local treatment	21 (87.5%)
Gene mutation status	
RAS and BRAF wild-type	5 (20.8%)
K-RAS mutant	16 (66.7%)
BRAF mutant	1 (4.2%)
Unknow	2 (8.3%)
MMR or MSI status	
pMMR or MSS	19 (79.2%)
dMMR or MSI-H	1 (4.2%)
Unknown	4 (16.7%)

*Abbreviations*: *mCRC* metastatic colorectal cancer, *pMMR* mismatch repair-proficient, *MSS* microsatellite stable, *dMMR* mismatch repair-deficiency, *MSI-H* microsatellite instability-high

Regorafenib plus PD-1 inhibitors were given to 13 patients (54.2%) in third-line treatment and 11 patients (45.8%) in fourth-line treatment and beyond. The most common PD-1 inhibitors were sintilimab (50.0%), followed by carrelizumab (25.0%), nivolumab (8.3%), toripalimab (8.3%), pembrolizumab (4.2%) and tislelizumab (4.2%). There were eight patients (33.3%) who previously received regorafenib therapy, and no one had taken PD-1 inhibitors before entering this study. MSI/MMR status data were available from 20 patients (83.3%). Of these, only one patient (4.2%) had an MSI-high tumor, and the remaining 19 patients (79.2%) were MSS or MMR proficient.

### Primary efficacy

**Fig. 1:**
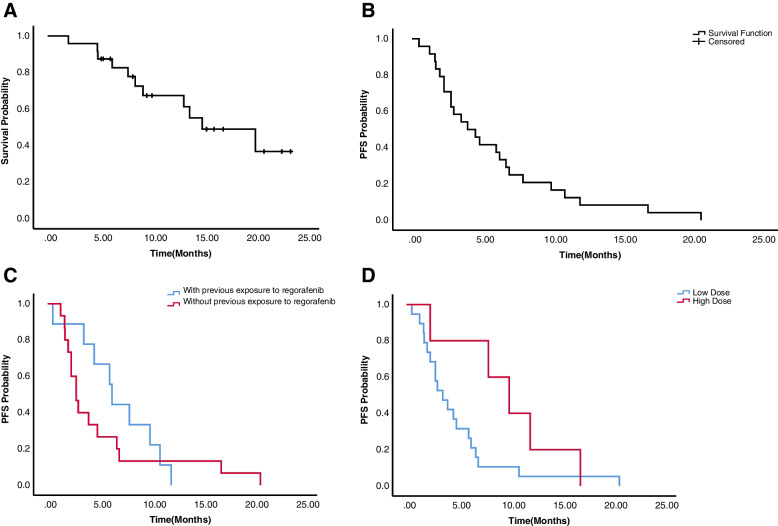
Kaplan–Meier survival curves. **A** The median OS was 15.0 months(95% CI, 7.0–23.0). **B** The median PFS was 4.0 months (95% CI, 1.8–6.2). **C** The median PFS for patients with or without previous exposure to regorafenib (6.3 months vs 2.8 months) (*P* = 0.445). **D** The median PFS for patients with final high daily doses group (regorafenib > 80 mg) and low group(regorafenib ≤ 80 mg) (10.0 months vs 3.5 months) (*P* = 0.106)

The median OS and median PFS were 15.0 months(95% CI, 7.0–23.0) and 4.0 months (95% CI, 1.8–6.2), respectively (Fig. 1A&B). Overall, the ORR and DCR were 8.3% and 70.8%, respectively. CR, PR, SD, PD, and patients without tumor response assessment were observed in 0, 2(8.3%), 15 (62.5%), 4 (16.7%), and 3 (12.5%) patients, respectively, as shown in Table 2.

**Table 2:** Characteristics of individual mCRC patients retrospectively analyzed in this study

	Age- ranges (year)	Sex	ECOG PS	Primary tumor location	Sites of metastasis when on treatment	KRAS /NRAS /BRAF mutation status	MMR or MSI status	Response and duration on prior Rego (mo)	Combining regimen	No. of cycles	Response	Regorafenib initial daily dose(mg)	Regorafenib final daily dose(mg)
1	61–65	F	1	Left	Liver	KRAS Mt	Unknown	PD (2)	Rego + Cam	3	SD	120	80
2	61–65	M	0	Right	Liver, lung	Wt	MSI-H	No Prior Rego	Rego + Sin	16	SD	80	40
3	66–70	M	1	Left	Lung,bone,lymph nodes, peritoneum	KRAS Mt	MSS	PD (2)	Rego + Pem	6	SD	80	80
4	61–65	M	1	Left	Liver, lung, lymph nodes, peritoneum	KRAS Mt	MSS	No Prior Rego	Rego + Sin	4	SD	80	80
5	71–75	M	1	Right	Liver, lung	KRAS Mt	MSS	No Prior Rego	Rego + Tori	9	SD	80	80
6	61–65	F	1	Left	Liver, lung, lymph nodes	Unknown	Unknown	No Prior Rego	Rego + Sin	2	PD	80	80
7	61–65	F	1	Left	Liver, lung	KRAS Mt	MSS	No Prior Rego	Rego + Sin	4	SD	80	80
8	61–65	F	0	Left	Lung	Wt	MSS	SD (1)	Rego + Cam	1	Unknown	120	80
9	71–75	M	0	Left	Liver	KRAS Mt	MSS	SD (6)	Rego + Sin	13	SD	80	160
10	66–70	M	1	Right	Lung, lymph nodes	KRAS Mt	MSS	No Prior Rego	Rego + Cam	4	PR	80	80
11	61–65	F	2	Right	Lymph nodes, peritoneum	Wt	Unknown	No Prior Rego	Rego + Tisle	1	Unknown	80	80
12	66–70	F	1	Right	Peritoneum	KRAS Mt	MSS	No Prior Rego	Rego + Nivo	3	SD	40	80
13	66–70	M	1	Right	Liver, lymph nodes	BRAF V600E Mt	MSS	SD (1)	Rego + Sin	13	SD	80	160
14	66–70	F	1	Left	Lung	KRAS Mt	MSS	No Prior Rego	Rego + Sin	2	PD	80	80
15	66–70	F	1	Left	Liver, lung,bone	KRAS Mt	MSS	No Prior Rego	Rego + Sin	3	SD	80	80
16	66–70	F	1	Left	Liver, lung,lymph nodes	KRAS Mt	MSS	No Prior Rego	Rego + Cam	2	SD	80	80
17	76–80	F	1	Left	Liver	KRAS Mt	Unknown	No Prior Rego	Rego + Tori	6	SD	80	80
18	66–70	F	1	Left	Liver	Unknown	MSS	SD (8)	Rego + Cam	13	SD	120	120
19	66–70	M	2	Right	Lung,bone,brain	KRAS Mt	MSS	No Prior Rego	Rego + Cam	1	Unknown	80	80
20	71–75	F	2	Left	Liver, lung,lymph nodes	KRAS Mt	MSS	No Prior Rego	Rego + Sin	2	PD	80	40
21	66–70	F	0	Left	Lung, lymph nodes	KRAS Mt	MSS	SD (2)	Rego + Sin	15	SD	80	80
22	66–70	F	1	Left	Liver, lung,lymph nodes	Wt	MSS	No Prior Rego	Rego + Nivo	2	PD	80	40
23	66–70	M	1	Right	Liver	Wt	MSS	PD (6)	Rego + Sin	6	SD	80	80
24	61–65	F	0	Left	Lung, lymph nodes	KRAS Mt	MSS	No Prior Rego	Rego + Sin	12	PR	120	160

*Cam* camrelizumab, *ECOG PS*, Eastern Cooperative Oncology Group performance status, *F* female, *M* male, *mo* months *Mt* mutant, *MSI-H* microsatellite instability high, *MSS* microsatellite stability, *Nivo* nivolumab, *Pem* pembrolizumab, *PD* progressive disease, *Rego* regorafenib, *PR* partial response, *SD* stable disease, *Sin* sintilimab, *Tisle* tislelizumab, *Tori* toripalimab, *Wt* wild-type

Specifically, patients previously treated with regorafenib had longer median PFS than those without it. Median PFS was 6.3 months (95% CI, 5.6–7.0) in the former and 2.8 months (95% CI, 1.9–3.7) in the latter (*P* = 0.445, Fig. 1C). No significant associations for PFS could be seen in whether previously treated with bevacizumab (*P* = 0.874), K-RAS status (*P* = 0.150), tumor sites (*P* = 0.321), and any kind of PD-1 inhibitors (*P* = 0.477).

16.6% of patients (*n* = 4) started regorafenib at daily doses of 120 mg, 79.2% of patients (*n* = 19) started at 80 mg, and 4.2% of patients (*n* = 1) started at 40 mg. There were 12.5%, 70.8%, 4.2%, and 12.5% of patients who received the final daily doses of 40, 80, 120, and 160 mg, respectively. Dose modifications were performed in 9 overall patients (37.5%), including dose reduction in 5 patients (20.8%) and dose escalation in 4 patients (16.7%) (Table 2). Furthermore, median PFS and OS in final high-dose group (daily dose above 80 mg of regorafenib) had better trends than those in final low-dose group (daily dose no more than 80 mg of regorafenib). Median PFS was increased in final high daily doses group (10.0 months) vs (3.5 months) with final low daily doses group (Fig. 1D). Median OS was also increased in the final high-dose group (not reach) versus the final low-dose group (15.0 months).

### Safety

Five patients had a dose reduction due to adverse events, hand-foot skin reaction in 3 patients, hypertension in 1 patient, and proteinuria in 1 patient. The most common adverse events were hand-foot skin reaction, fatigue, hypertension, and diarrhea. The grade 3 to 4 adverse events occurred in 5 patients (20.8%). The most common grade 3 to 4 events were hand-foot skin reaction, hypertension, and oral mucositis.

## Discussion

As the majority of CRC cases display a molecular MSS/pMMR profile, it is particularly meaningful to investigate the clinical applications of adaptive immune or combination regimens in MSS CRC patients. Several studies have shown that the combination regimen using regorafenib combined with PD-1 inhibitors has promising efficacy for those patients [12–18]. The highest ORR, which can be seen in the REGONIVO study, was 33%; and the median OS from REGOTORI was up to 15.5 months. However, there is a lack of clear evidence regarding the real-world effects of this therapeutic strategy, especially in elderly Chinese patients.

In this single-center, retrospective study involving Chinese elderly patients with refractory advanced colorectal cancer, the median age of patients enrolled in our study is 68 years, which is older than that in most studies conducted with regorafenib or regorafenib plus PD-1 inhibitors (53.0 to 61.3 years) [4, 5, 12, 14, 15]. Notably, the reported median OS of 15.03 months was comparable to those reported in REGOTORI (15.5 months) [13]. The combination using regorafenib plus PD-1 inhibitors seemed to achieve a better OS and ORR (8.3%) than regorafenib alone (ORR 1%–4%; OS 6.4–8.8 months) for patients with mCRC [4, 5]. Although the combination regimen yields a response rate of less than 10%, which is lower than those reported in REGOTORI (15.2%) [13] and REGONIVO (36.0%) [12], more than 70% of patients (70.8%) in our study achieved disease control, which was superior to the results of single-agent regorafenib, and similar to those from another China trial [4, 5, 14]. Hence, there is a possible reason that patients who achieved higher DCR resulted in better OS in our study. Overall, these results showed that it was worth to be recommended regorafenib plus PD-1 inhibitors for elderly patients with mCRC, especially in patients with a molecular MSS/pMMR profile.

Further analyses of effectiveness in our study showed that there was no obvious correlation for survival between patients who only had liver metastasis and lung metastasis (*P* > 0.05). In addition, PFS was similar for patients with KRAS wild-type versus mutant, left- versus right-sided tumors, and among various types of PD-1 inhibitors. However, patients with previous treatment with regorafenib had longer PFS than those without it (6.30 months versus 2.80 months, *P* > 0.05). Several reports showed that regorafenib modulated immuno-suppressive tumor microenvironment by blocking VEGFRs, TIE2, and CSF-1R, RET/Src axis signal pathways, and increased intratumoral CD45^+^ leukocytes, CD8^+^ T cells, which would enhance anti-tumor immunity when using regorafenib alone and plus various immunotherapies [19–21]. This could be why patients who had previously received regorafenib obtained longer PFS. Further resources and research should be conducted to address the time of administration for regorafenib plus PD-1 inhibitors.

We noticed that most patients in our cohort started at the lower dose of regorafenib. All were at doses of less than 120 mg, and patients started at these of ≤ 80 mg, accounting for 83.4%. During follow-up, four patients received high-dose, and five patients had doses reduction. Consequently, the number of patients who received less than 80 mg doses stayed the same as those in baseline, whereas four patients (16.7%) raised the doses up to 120 mg or 160 mg. When compared with PFS and OS in different initial daily dose groups of regorafenib (≤ 80 versus > 80 mg), we concluded that there was no significant difference between these groups. And similar results were found in the final daily doses groups. However, it showed a trend toward better PFS in patients with final daily doses > 80 mg group compared to the low-dose group (median PFS was 10.0 months in the high-dose group versus 3.5 months in ≤ 80 mg). Considering the safety and tolerability profile of regorafenib, we argue that it should be started with a lower dose for regorafenib and adjusted until the maximum tolerated dose was reached by periodic follow-up and communication in time between nurses and patients. This strategy could contribute to enhancing tolerability and improving adherence, and reducing the risk of adverse events from regorafenib and/or PD-1 inhibitors.

The limitations of our study include its retrospective nature, the inadequate data on toxicity evaluation, the small sample size, and the short median follow-up. Besides, there were 4 PD-1 inhibitors just approved in China but no other countries, which means these therapeutic drugs are not available in other countries. However, the results may provide a clearer picture of the efficacy and safety of regorafenib plus PD-1 inhibitors in elderly Chinese patients with mCRC.

To our knowledge, this is the first paper describing the detailed information on regorafenib plus PD-1 inhibitors in elderly Chinese patients with mCRC in real-world settings. This real-world evidence confirms that Chinese elderly patients with advanced colorectal cancer can benefit from the treatment of regorafenib combined with PD-1 inhibitors, similarly with this combination therapy strategies in all age patients. However, further larger cohorts research should investigate whether the PFS advantage in the high-dose group could eventually lead to improved OS outcomes. Besides, considering the quality of life, close monitoring and management for adverse drug events are needed.

## Data Availability

The datasets presented in this article are not readily available because of Chinese regulations and conditions for informed consent. Requests to access the dataset should be directed to Beibei Chan, zlyychenbb1429@zzu.edu.cn.
